# The Role of the Chemokine System in Tissue Response to Prosthetic By-products Leading to Periprosthetic Osteolysis and Aseptic Loosening

**DOI:** 10.3389/fimmu.2017.01026

**Published:** 2017-08-24

**Authors:** Tereza Dyskova, Jiri Gallo, Eva Kriegova

**Affiliations:** ^1^Faculty of Medicine and Dentistry, Department of Immunology, Palacky University Olomouc, Olomouc, Czechia; ^2^Faculty of Medicine and Dentistry, Department of Orthopaedics, Palacky University Olomouc, University Hospital Olomouc, Olomouc, Czechia

**Keywords:** chemokine receptors, tissue homeostasis, immunoregenerative implant, wear particles, aseptic loosening, osteolysis, therapeutics

## Abstract

Millions of total joint replacements are performed annually worldwide, and the number is increasing every year. The overall proportion of patients achieving a successful outcome is about 80–90% in a 10–20-years time horizon postoperatively, periprosthetic osteolysis (PPOL) and aseptic loosening (AL) being the most frequent reasons for knee and hip implant failure and reoperations. The chemokine system (chemokine receptors and chemokines) is crucially involved in the inflammatory and osteolytic processes leading to PPOL/AL. Thus, the modulation of the interactions within the chemokine system may influence the extent of PPOL. Indeed, recent studies in murine models reported that (i) blocking the CCR2–CCL2 or CXCR2–CXCL2 axis or (ii) activation of the CXCR4–CXCL12 axis attenuate the osteolysis of artificial joints. Importantly, chemokines, inhibitory mutant chemokines, antagonists of chemokine receptors, or neutralizing antibodies to the chemokine system attached to or incorporated into the implant surface may influence the tissue responses and mitigate PPOL, thus increasing prosthesis longevity. This review summarizes the current state of the art of the knowledge of the chemokine system in human PPOL/AL. Furthermore, the potential for attenuating cell trafficking to the bone–implant interface and influencing tissue responses through modulation of the chemokine system is delineated. Additionally, the prospects of using immunoregenerative biomaterials (including chemokines) for the prevention of failed implants are discussed. Finally, this review highlights the need for a more sophisticated understanding of implant debris-induced changes in the chemokine system to mitigate this response effectively.

## Introduction

Nowadays, millions of joints are being replaced worldwide and the number is gradually increasing. Although total joint replacement (TJR) represents one of the most successful procedures in all of medicine, it may be complicated by numerous complications, periprosthetic osteolysis (PPOL) and aseptic loosening (AL) being the ones most frequently seen in the long-time horizon (1). According to current hypothesis, wear particles liberated from the bearing surface of implants activate immune, inflammatory, and resident tissue cells to release various inflammatory mediators and regulatory molecules, chemotactic cytokines (chemokines) being the most prominent among them (2–4). The continuous release of chemokines, cytokines, and other mediators promotes an inflammatory microenvironment in which osteoclasts are stimulated, specifically by the receptor activator of the nuclear factor-kappaB (RANK) ligand. All these processes contribute to bone resorption (i.e., PPOL), leading eventually to AL of the implant (5–7).

The current knowledge about the pathogenic role of the chemokine receptors and their ligands—chemokines (further referred to as the “chemokine system”) in PPOL/AL arises mainly from studies in animal models and cell lines related to wear particle-induced PPOL. In human PPOL/AL, implant debris-induced changes within the chemokine system are incompletely characterized.

Currently, chemokine receptors are being intensively studied as promising therapeutic targets in various bone-associated pathologies (8–10). Although such research in wear particle-induced osteolysis is in its infancy, the prospects of targeting the chemokine system to prevent PPOL/AL are evident from animal models of osteolysis (11–13). Moreover, chemokines incorporated into implant surfaces or embedded in hydrogels on implant surfaces have been shown to promote tissue regeneration, regulate the recruitment of inflammatory cells, and attenuate osteolysis (14–16), thus leading to lower rates of reoperations resulting from PPOL/AL.

In this review, we summarize the current knowledge on the role of the chemokine system in PPOL/AL. Moreover, we discuss the potential for mitigating the osteolytic processes after the modulation of the chemokine system interactions and/or the implication of implants with immunoregenerative surfaces for preventing premature prosthesis failure of artificial joints.

## The Chemokine System and Its Role in the Periprosthetic Microenvironment

**Figure 1:**
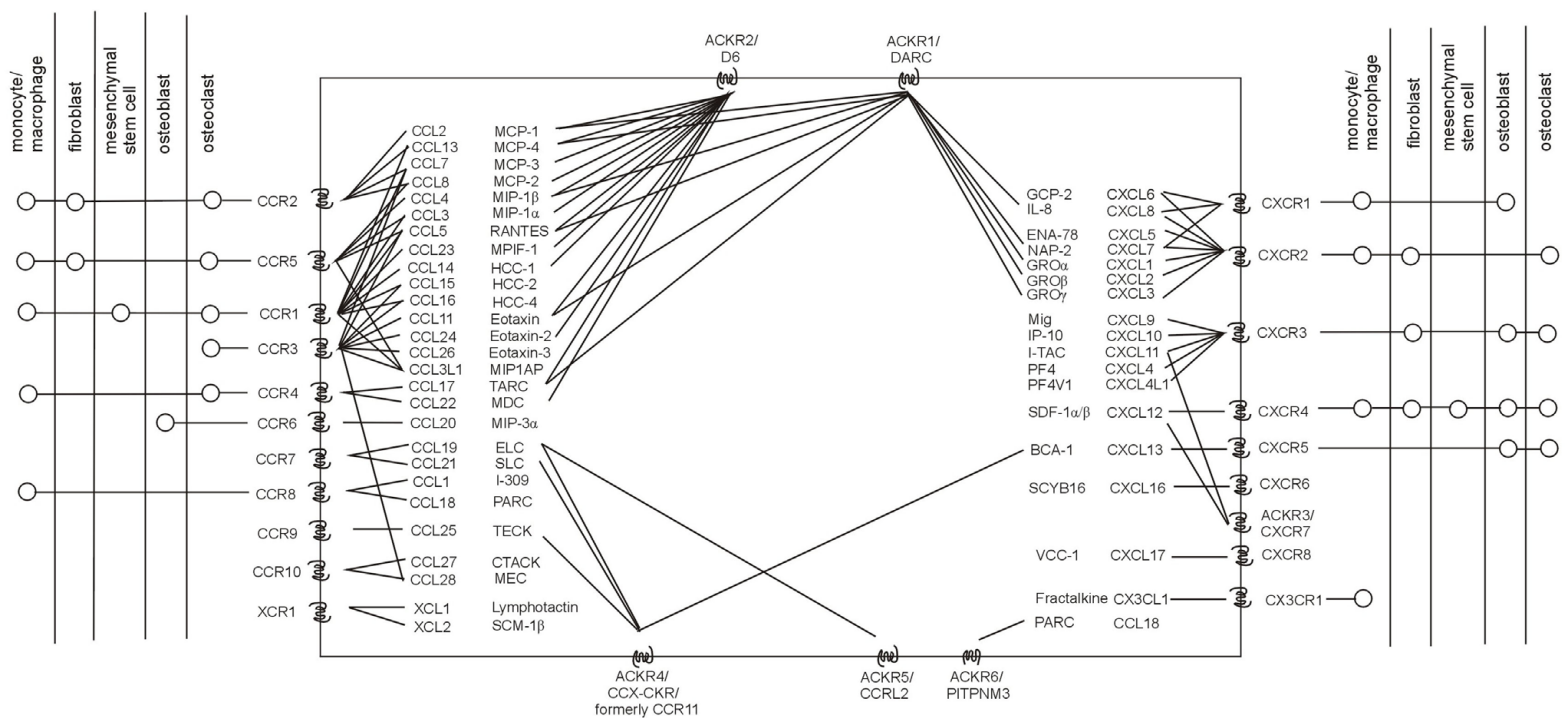
Human chemokine system. Chemokine receptors with their known ligands (incl. their systematic and trivial names), as well as the current knowledge on the presence of chemokine receptors in major cell subpopulations related to osteolysis are stated. Adapted from Ref. (20, 22, 30).

Chemokine receptors are members of the class of seven-transmembrane G protein-coupled receptors (17). The chemokine receptor family consists of 19 members divided into several classes according to their ligands, chemokines (Figure 1) (18, 19). In addition, six atypical (non-chemotactic, recycling, or scavenging) chemokine receptors have recently been described (20–22). The interaction between chemokine receptors and their ligands, chemokines, triggers the cascade of downstream signaling, leading to various biological functions (23–26). For more details on the chemokine system, see recent review articles (27–29).

The major role of the chemokine system relevant to the context of orthopedic implant pathology includes cell trafficking of immune and inflammatory cells from circulation to the bone–implant interface. Besides its contribution to cell migration (3), the chemokine system also participates in apoptosis, angiogenesis, tissue repair, and regeneration (28, 31, 32), as well as in the production of collagen (33). Because of the limited data on other functions of the chemokine system at the bone-implant interface, further studies are needed.

So far, only limited data exist on the increased gene expression of chemokines, namely CXCL8 (34), CCL3 (34, 35), and CCL2 (36), in periprosthetic tissues from aseptically loosened implants. Protein studies profiling inflammatory cytokines/chemokines on tissues from patients with end-stage PPOL showed elevated CXCL8, 9, and 10 and IL-6 but no IL-1 or TNF (37). These authors also suggest that CXCL8 and IL-6 may be the primary drivers of osteoclastogenesis. In addition, elevated CXCL8 expression correlated with early time to revision (38). Moreover, the elevation of the chemokines CXCL9 and CXCL10 was reported in adverse local tissue reactions associated with tribocorrosion following total hip arthroplasty (39).

Considering chemokine receptors, further studies are needed to elucidate their role in PPOL/AL. However, there is already evidence from other bone-related diseases (40, 41) and cell lines in experimental osteolysis (42, 43) that chemokine receptors are present in all the major subpopulations involved in the pathogenesis of wear particle-induced osteolysis, such as monocytes/macrophages, giant cells, osteoclasts, osteoblasts, fibroblasts, dendritic cells, lymphocytes, and mesenchymal stem cells (MSCs) (Figure 1). One may, therefore, deduce that chemokine receptors and their ligands expressed on these osteolysis-associated cells in response to wear particles and/or cytokines involved in PPOL/AL pathogenesis may potentially also contribute to human PPOL/AL, thus deserving further investigation. The formation of receptor dimers and oligomers at the cell surface (44), which can modify the chemokine binding and signaling activity, as well as the complexity and redundancy of the chemokine system, should also be taken into account (45).

## Chemokine Receptors on Osteolysis-Associated Cells

Macrophages are the major cells in host defense, responding to wear particles *via* the production of cytokines and chemokines and, second, as precursors for osteoclasts responsible for ensuing bone resorption (6, 7). Murine macrophage-like (RAW) cells have been shown to express the chemokine receptor CCR1, and its ligands, the chemokines CCL3, CCL5, and CCL7, were able to stimulate the chemotaxis of RAW cell precursors (46). Murine RAW cells also express CXCR2, and its expression increases after RANKL treatment (13). A ligand of CCR2, chemokine CCL2, mediates the systemic migration of murine macrophages in the presence of continuous particle infusion (12). Additionally, CXCR4 is highly expressed by human monocytes (47), and its ligand, CXCL12, markedly stimulates the chemotactic recruitment of circulating human monocytes capable of generating bone-resorptive osteoclasts (47). A study of primary human macrophages challenged with various stimuli showed the elevation of the cytokines IL-1α, TNF-α, and IL-1β and chemokines CCL2 and CXCL8 but not CXCL9 or CXCL10 (48). Importantly, TiAlV particles were the most stimulatory, followed by CoCr and alumina particles; polyethylene debris did not stimulate human macrophages to secrete cytokines (48). In contrast, there is evidence for polyethylene particles inducing the expression of inflammatory cytokines (49–51). If the level of polyethylene particles in the periprosthetic environment is taken into account (52), these are the most detrimental by-products liberated from TJR.

Regarding human fibroblasts, their exposure to titanium and polymethylmethacrylate (PMMA) particles resulted in the increased release of CCL2 in a dose- and time-dependent manner (53). In addition, IL-1β stimulated the release of CCL2, CCL8, and CCL5 from the fibroblasts (53). The stimulation of human fibroblasts with wear debris resulted in the upregulated secretion of CCL2, IL-1β, IL-6, IL-8, TGF-β1, and TGF-β receptor type I, as well as matrix metalloproteinase 1, cyclooxygenase-1 and -2, and leukemia inhibitory factor 1 (54). Moreover, studies in patients with rheumatoid arthritis (RA) showed that fibroblast-like synoviocytes (FLS) constitutively express CCR2, CCR5, CXCR3,and CXCR4; in addition, stimulation with CCL2, CXCL12, CXCL9, and CXCL10 enhances FLS migration and proliferation (55, 56). Furthermore, the upregulated expression of CCR3 in FLS from RA patients is induced by CCL11 (57). Moreover, CCR7 is expressed on the FLS of patients with RA and osteoarthritis (OA) (58). FLS migrated in response to the CCR7 ligands CCL19 and CCL21 and the stimulation of FLS with CCL19 resulted in a markedly increased secretion of vascular endothelial growth factor of RA- and OA-FLS (58). Finally, FLS secretes joint fluid into the joint capsule under both normal and pathological conditions. In relation to PPOL/AL, joint fluid waves undoubtedly contribute to an implant–bone pathology (59). However, the exact role of the chemokine system in the wear-induced production of joint fluid remains to be elucidated. Considering fibrocytes, which have both the inflammatory features of macrophages and the tissue-remodeling properties of fibroblasts, limited information on the expression of the chemokine system and its involvement in PPOL/AL exists (6, 60).

Human osteoblasts express CXCR1 and CCR6 (61), and their ligands, CXCL8 and CCL20, strongly enhance osteoblast-mediated osteoclastogenesis through the upregulation of IL-6 production by osteoblasts (61). Furthermore, stimulation with Ti particles increased the expression of the CCR4 ligands CCL17 and CCL22 in human osteoblasts (62). In addition, human osteoblasts express CXCR3, CXCR4, and CXCR5 (63, 64). CXCR4 expression in human osteoblast-like cells is induced by CoCr particles (65). Primary bone marrow preosteoclast populations are positive for the CCR1 receptor, and its expression increases markedly during RANKL-induced osteoclast formation (46).

Mesenchymal stem cells represent another cell population crucially involved in PPOL/AL. In human MSCs, the CCR1 ligand CCL3 promotes chemotaxis to PMMA particles (66). In the murine model of PPOL, the CCR1 receptor mediates the systemic migration of MSCs in the presence of polyethylene wear particles (67). In addition, stimulation with Ti particles increased CXCL8 expression in MSCs (68).

Moreover, primary bone marrow preosteoclasts express CCR2, CCR3, and CCR5 (46). Human osteoclasts are positive for the CCR1, CCR2, CCR3, and CCR4 receptors (69, 70), while CCR2 and CCR4 are potently induced by RANKL and CCL2 (71). Additionally, stimulation with Ti particles increased the expression of the CCR4 ligands CCL17 and CCL22 in human osteoclasts (62). In addition, human osteoclasts grown *in vitro* express CXCR3, CXCR4, and CXCR5 (72). CXCR4 is also highly expressed by osteoclasts generated *in vitro*, osteoclast-like cells, and mature osteoclasts isolated from human femoral bones (47). The CXCR4 ligand CXCL12 directly promotes the early stages of osteoclast development after M-CSF/RANKL treatment *via* stimulating precursor cell numbers, multinucleated cell fusion, and increased cell size (47). Considering osteocytes, cells actively involved in the bone matrix turnover and bone resorption through various mechanosensory mechanisms, limited information on the expression of the chemokine system and their involvement in PPOL/AL exists (73–75). The reasons are mainly the methodological obstacles related to their investigation.

## Animal Models for PPOL

**Figure 2:**
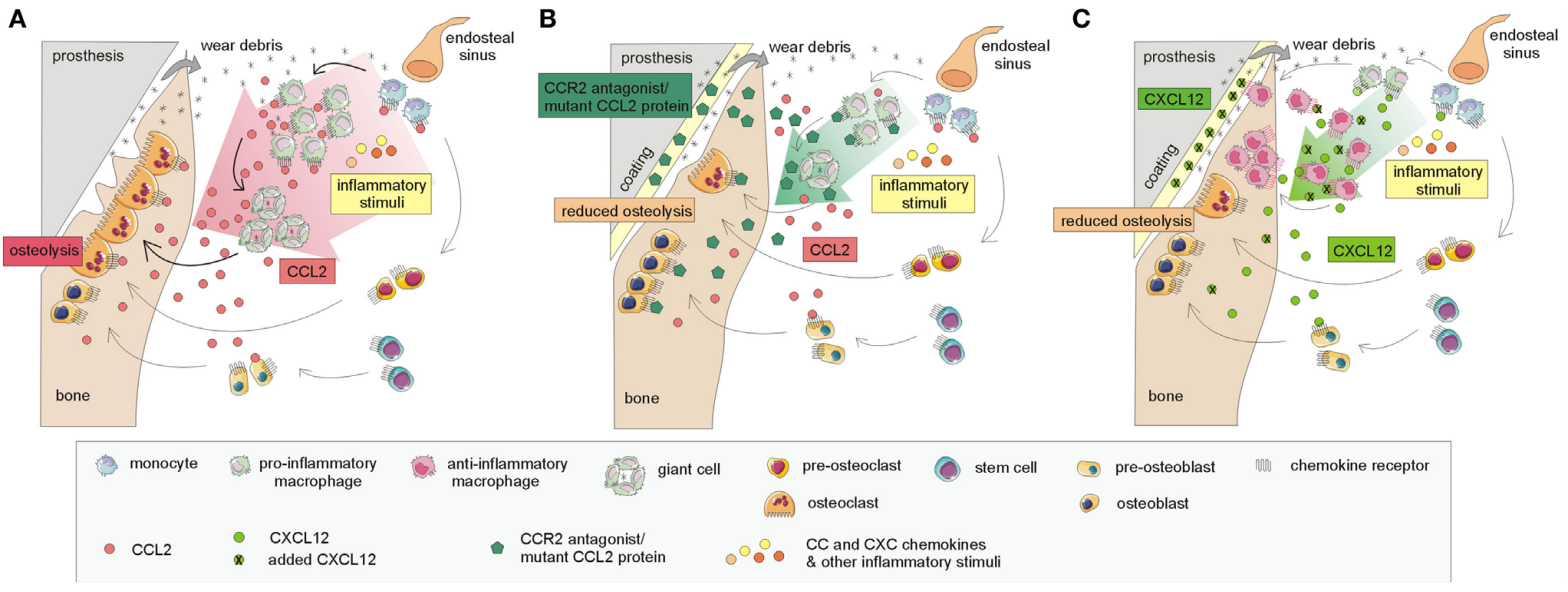
Currently known mechanisms involved in targeting the CCR2–CCL2 and CXCR4–CXCL12 interactions based on murine models leading to the attenuation of the osteolytic process. **(A)** A simplified model of the host response to prosthetic by-products: CCL2 chemokine and others, released in response to implant wear debris, induce the recruitment of macrophages and other immune cells to the implant surroundings, thus inducing inflammation and osteolysis at the bone–implant interface. The interaction of CCL2 with its receptor CCR2 and others further enhances the recruitment of macrophages and other immune cells and stimulates the formation of giant cells, differentiating into osteoclasts, which are responsible for bone resorption. **(B)** Incorporation of CCR2 antagonist/mutant CCL2 protein into hydrogel on the implants: the release of the CCR2 antagonist/mutant CCL2 protein results in the reduction of macrophage recruitment to the implant surroundings, reduced giant cell/osteoclast formation, and reduced osteolysis. **(C)** Incorporation of CXCL12 on implant surface: the incorporation of CXCL12, attached to the implant surface, may also help to reduce osteolysis. Macrophages recruited to the implant surroundings interact through the CXCR4 receptor with administered CXCL12; this interaction leads to the polarization of macrophages toward anti-inflammatory "healing" phenotypes, contributing to bone regeneration.

Current findings based on cell lines and murine models point to several receptor–chemokine interactions, namely CCR1–CCL3, CCR2–CCL2, CXCR2–CXCL2, and CXCR4–CXCL12, which appear to be crucially involved in osteolysis. The most central role has been suggested to be that of the CCR2–CCL2 axis; other receptor–chemokine axes such as CCR1–CCL3, CXCR2–CXCL2, and CXCR4–CXCL12 have a less clear role in implant debris-induced inflammation (Figure 2). For these receptor–chemokine pairs, initial data are already available on targeting these axes in *in vitro* and/or *in vivo* studies in animal models (11–13, 67) (Table 1). Other receptor–chemokine interactions have not yet been elucidated in PPOL; however, initial data exist on other bone-related diseases (8–10). Nevertheless, it should be taken into account that the data obtained may not reflect the widespread expression of some chemokine receptors, thus not predicting the overall consequences of receptor inhibition.

**Table 1:** Targeting of chemokine receptors and its effect in osteolytic experimental animal models.

Chemokine receptor	Treatment/condition	Experimental model	Outcome	Reference
CCR1	CCR1 antagonist (J-113863)	Murine model of continuous local infusion of UHMWPE particles	Reduced MSC chemotaxis, more profound adverse effects on bone mineral density	Gibon et al. (67)

CCR2	CCR2 antagonist (RS102895)	Murine model of continuous local infusion of UHMWPE particles	Decrease of systemic migration of macrophages	Gibon et al. (12)
	CCR2 deficiency	Murine model of continuous local infusion of UHMWPE particles, injection of CCR2 −/− macrophages	Lower macrophage recruitment	Gibon et al. (12)
	Mutant CCL2 protein (7ND)	Wear particle-induced osteolysis in murine calvarial model	Reduced wear particle-induced osteolysis, higher bone volume fraction, decrease of recruited inflammatory cells and osteoclasts	Jiang et al. (76); Nabeshima et al. (16)

CXCR2	siRNA targeting CXCR2	Ti-induced osteolysis in mouse calvarial model	Inhibition of osteolysis, suppression of osteoclast formation	Wang et al. (13)

CXCR4	CXCR4 antagonist (T140)	Murine model of multiple myeloma-mediated osteolysis	Reduced osteoclast recruitment, lower migration of osteoclast precursors	Diamond et al. (11)
	CXCR4 deficiency	Murine model of bone metastasis	Elevated markers of bone resorption, increased bone loss	Hirbe et al. (77)
	CXCL12 administration	Murine implantation model	Reduced inflammatory and fibrotic response, increased angiogenesis	Thevenot et al. (14)

*MSC, mesenchymal stem cells; UHMWPE, ultra-high-molecular-weight polyethylene*.

Additionally, current promising results based on murine models of osteolysis need to be considered with circumspection, since these models only imitate the real situation in humans with TJR. The majority of studies are performed on non-implant models, i.e., models of wear particle-induced osteolysis, independent of the critical biomaterial and biomechanical components (78, 79). Furthermore, the differences between humans and mice in terms of lifespan, as well as differences in gait and weight, which influence the load and size of the wear particles that are released, need to be taken into account. Another weakness is related to the time axis of osteolysis: the murine models available represent an acute rather than chronic disease since the osteolytic processes in these models are induced within days or weeks, whereas in humans, months and years should be considered.

## Targeting the CCR2–CCL2 Axis

There is a growing body of evidence about the central role of the CCR2–CCL2 axis in PPOL/AL. Implant debris can induce the production of CCL2 in human fibroblasts, osteoblasts, monocytes, and macrophages (Figure 1), leading to the chemoattraction of monocytes, macrophages, and NK and T cells (80, 81). There is already evidence from *in vivo* studies that blocking the CCR2–CCL2 pathway may attenuate the osteolysis, with several different mechanisms probably involved. In a murine femoral implant model, the blocking of CCR2–CCL2 reduced macrophage recruitment to the site of the implant (12). In contrast, an *in vitro* study did not confirm that blocking CCR2–CCL2 interaction is effective in blocking macrophage recruitment (66). Another *in vivo* study reported that blocking CCL2 disrupted the formation of osteoclast-like multinuclear cells, thus blocking bone resorption (71). Recombinant protein 7ND, a mutant of CCL2 that inhibits CCR2 signaling, has also been shown to effectively reduce macrophage migration, the number of osteoclasts, and wear particle-induced bone loss when incorporated into the implant coating (16, 76).

Despite the centrality of the CCR2–CCL2 axis, it seems unlikely that the interruption of only this pathway may prevent PPOL, mainly because of the pleiotropic nature of the chemokine system. CCL2 binds to CCR2, but on the other hand, CCR2 is also able to interact with CCL7, CCL8, and CCL13. The contribution of other CCRs and chemokines to the recruitment of macrophages to the site of the implant has already been proven in a study with injected CCR2-deficient macrophages (82).

## Targeting the CCR1–CCL3 Axis

The CCR1–CCL3 axis is also heavily involved in particle-induced PPOL. CCL3 is produced mainly by macrophages, NK cells, fibroblasts, and mast cells, and its receptor CCR1 is present on monocytes, macrophages, osteoclasts, neutrophils, T and NK cells, and MSCs (Figure 1) (83, 84). Importantly, it seems that CCR1–CCL3 is a central mediator involved in the migration of MSCs to the sites of peri-implant inflammation. Indeed, treatment with a CCR1 antagonist in a murine model of continuous local infusion of appropriate polyethylene particles resulted in decreased MSC chemotaxis and more profound adverse effects on bone mineral density (67). Bone marrow-derived MSCs have the ability to differentiate into osteoblasts and produce osteoprotegerin, a decoy receptor for RANKL, naturally (85). The MSCs are positive for the CCR1 receptor, and its expression increases markedly during RANKL-induced osteoclast formation (46). In contrast, CCL3 has been shown to induce the differentiation of monocytes to osteoclasts and higher levels were found in osteolytic lesions around the implant (86). Therefore, it seems that the function of CCL3 in PPOL is dose, site, and time dependent. Currently, there is insufficient evidence to indicate CCR1–CCL3 for targeting in implant debris-induced inflammation and osteolysis. Moreover, the fact that CCL3 binds to CCR5 and CCR1 interacts with other potent chemokines such as CCL5, CCL7, CCL13, CCL14, CCL15, CCL16, and CCL23, as well as its presence in various osteolysis-related cells (Figure 1), should also be taken into account.

## Targeting the CXCR4–CXCL12 Axis

The CXCR4–CXCL12 appears as another important axis in osteolysis. CXCR4 is expressed by a large number of osteolysis-associated cells, including osteoclasts, osteoblasts, fibroblasts, macrophages, and MSCs (Figure 1). Moreover, its expression is upregulated in the presence of metallic wear debris *in vitro* and *in vivo* (87). Its sole ligand, CXCL12, is a crucial chemoattractant and survival factor for osteoclastic cells (47, 88). Indeed, disruption of the CXCR4–CXCL12 interaction using the CXCR4 antagonist T140 resulted in decreased osteoclast recruitment and lower migration of osteoclast precursors, thus reducing bone resorption in a mouse model of multiple myeloma-mediated focal osteolysis (11). Another study by Hirbe et al. contrasts these findings: CXCR4-deficient mice exhibited elevated markers of bone resorption and increased bone loss, thus suggesting that the CXCR4 axis may regulate osteoclast formation and activity negatively (77). In concordance with these findings, CXCL12, when incorporated into poly lactic-co-glycolic acid scaffolds, reduces the inflammatory response, increases angiogenesis, and reduces fibrotic responses, thus improving tissue responses to an implant (14). Furthermore, CXCL12 was shown to contribute to accelerated wound closure and shifting the balance toward M2 “healing” macrophages (15). Despite these discordant observations, the CXCR4–CXCL12 axis seems to be crucially involved in osteoclast formation and osteolysis. However, its role needs to be considered contextually in a dose-dependent manner and in relation to the presence of other osteoclast-activating factors.

## Targeting the CXCR2–CXCL2 Axis

The CXCR2 receptor has also been shown to play a role in osteolysis. Its expression was confirmed on macrophages, where it may be increased by treatment with RANKL, the crucial osteoclast differentiation factor (13). Its ligand, CXCL2, enhances the proliferation of osteoclast precursor cells and induces osteoclast formation (89). The inhibition of CXCR2 seems to be promising for the treatment of PPOL/AL since the local injection of adenovirus-mediated siRNA targeting CXCR2 inhibited Ti-induced osteolysis in a mouse calvarial model (13). Moreover, the administration of siRNA targeting CXCR2 suppressed osteoclast formation, both directly by acting in osteoclasts and indirectly by altering RANKL and OPG expressions in osteoblasts *in vitro* (13). Given these findings, CXCR2/CXCL2 also appears to be a possible therapeutic target for the prevention of osteolysis; however, additional studies focused on this axis are required.

## Targeting Other Receptor–Chemokine Axes

Other chemokine receptors such as CXCR3 and CX3CR1 may also represent promising therapeutic targets, as shown in various bone-affecting disorders and/or their animal models (8, 9). However, there is limited information on their involvement in PPOL/AL, which thus deserves further investigation. Current findings show that the blockade of the CX3CR1–CX3CL1 axis by the anti-CX3CL1 antibody strongly inhibited the osteoblast-induced differentiation of osteoclasts *in vitro* and led to a decreased number of mature osteoclasts actively resorbing the bone *in vivo* (8). Regarding CXCR3, its blockade reduced the severity of joint inflammation in arthritic animals through the inhibition of neutrophil accumulation in the joints, low leukocyte infiltration of the synovium, and loss of articular cartilage in the joints (9). Since the CXCR3 ligands CXCL9 and CXCL10 are elevated in PPOL/AL (37), the interaction between CXCR3 and these ligands should be investigated further. Besides the investigation of other chemokine receptor–chemokine axes in PPOL/AL, special emphasis should also be placed on other possible functions of the chemokine system, such as the regulation of bone regeneration and secretion of bone matrix proteins.

## Implants with Bioactive Surfaces Involving Chemokines

**Figure 3:**
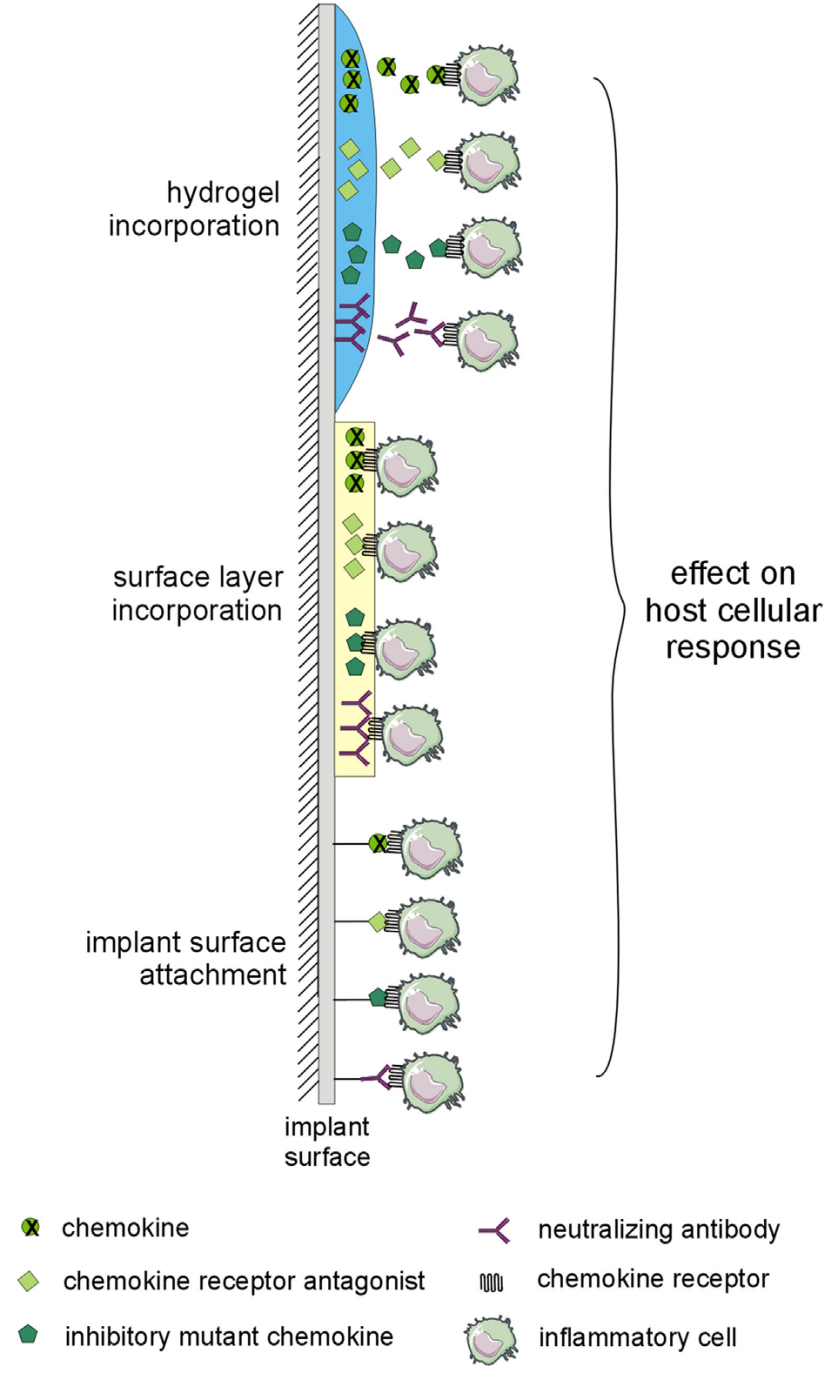
Implant modifications potentially utilizable for preventing adverse host reaction to implant by-products. The implant surface may be modified by layer-by-layer coatings or by hydrogels with incorporated chemokines, inhibitory mutant chemokines, antagonists of chemokine receptors, or neutralizing antibodies to the chemokine system. The changes in interactions within the chemokine system may affect the host cellular response to implant by-products.

There is a growing body of evidence about the potential of the application of chemokines, inhibitory mutant chemokines, antagonists of chemokine receptors, or neutralizing anti-chemokine receptors or anti-chemokine antibodies to the bone–implant interface in order to prevent an adverse reaction to TJR by the attenuation of cell recruitment and polarization of macrophages and tissue response generally, as well as the stimulation of microvascular network remodeling. Although several designs for the application of the desired molecules on the surface of the implant exist (Figure 3), those that are most studied are hydrogels with incorporated CXCL12 and mutant CCL2 protein.

It has been shown that hydrogels releasing CXCL12, a CXCR4 ligand, implanted in a murine dorsal skinfold window chamber promoted the spatially localized recruitment of anti-inflammatory monocytes and stimulated microvascular network remodeling (90). In addition, the dual delivery of chemokine CXCL12 together with sphingosine-1-phosphate accelerated wound closure; the combination of CXCL12 and SEW2871 (an agonist for the receptor S1P1) shifted the balance toward M2 “healing” macrophages (15). While local sphingosine-1-phosphate receptor 3 (S1P3) agonism recruits anti-inflammatory monocytes to remodeling vessels, the use of films delivering an agonist of S1P3, FTY720, to inflamed and ischemic tissues results in a reduction of pro-inflammatory cytokine secretion and an increase in regenerative cytokine secretion (91).

Additionally, it has been reported that the incorporation of CXCL12 into poly lactic-co-glycolic acid scaffolds reduced the inflammatory response, increased angiogenesis, and reduced fibrotic responses, and thus improved the response of the tissue to biomaterial implants (14). Keeney et al. developed a biodegradable coating allowing the efficient loading and controlled release of mutant CCL2 proteins (7ND) from the surface of orthopedic implants to block CCR2 signaling (16, 92). Mutant protein 7ND released from this coating retained its bioactivity and effectively reduced macrophage migration toward CCL2, the number of osteoclasts, and wear particle-induced bone loss. This strategy may thus be used to modulate anti-inflammatory responses and to prolong the lifetime of orthopedic implants (16, 92). However, the main problem that needs to be resolved is the development of a strategy for the appropriate time-dependent triggering of bioactive agents and their integration with the individual host’s response to the implant. Moreover, a hydrogel-based strategy, as well as biodegradable coatings designed to prevent PPOL/AL, has several limitations in terms of its short-term and generally unstable character in contact with the peri-implant environment.

Some studies have already shown that bone scaffolds can be designed to control the macrophage phenotype through the conjugation and release of immunomodulatory cytokines (IFN-γ promoting the M1 “pro-inflammatory” phenotype and IL-4 promoting the M2 “healing” phenotype), with resulting effects on scaffold vascularization (93). A similar observation was obtained using silk films with embedded IFN-γ- or IL-4-promoting M1 or M2 polarization, respectively (94). Other recent studies also highlighted the fact that the choice of “immunoregenerative” implants may control macrophage behavior and attraction, thus influencing the inflammation and repair processes (95–98). In contrast, the contribution of biomaterials in conjugation with immunomodulatory cytokines to the control of macrophages in terms of their immunophenotype, polarization, behavior, and attraction deserves much more research, and stronger evidence before the criteria for the approval of a clinical study are fulfilled.

Besides coating with chemokines, inhibitory mutant chemokines, antagonists of chemokine receptors, or neutralizing antibodies to the chemokine system as appropriate (Figure 3) (16, 90), the implant surface may also be modified by peptide sequences interacting with signaling proteins (99, 100), or collagen-mimetic peptides promoting cell adhesion and osteoblastic differentiation (101, 102).

## Therapeutic Prospects for the Chemokine System in PPOL/AL

Current findings based on cell lines and murine models point to several chemokine receptor–chemokine axes, namely CCR2–CCL2, CXCR2–CXCL2, and CXCR4–CXCL12, which show promising therapeutic prospects in the context of avoiding the side effects of orthopedic implant debris. However, the translation into pharmacological or biomaterial-driven strategies based on human studies investigating the role of the chemokine network during the time axis of PPOL/AL is very difficult. The major limitations of the systemic/local pharmacological interventions targeting the chemokine system may represent potential side effects since no single chemokine receptor or chemokine specific to PPOL/AL has been discovered so far.

**Figure 4:**
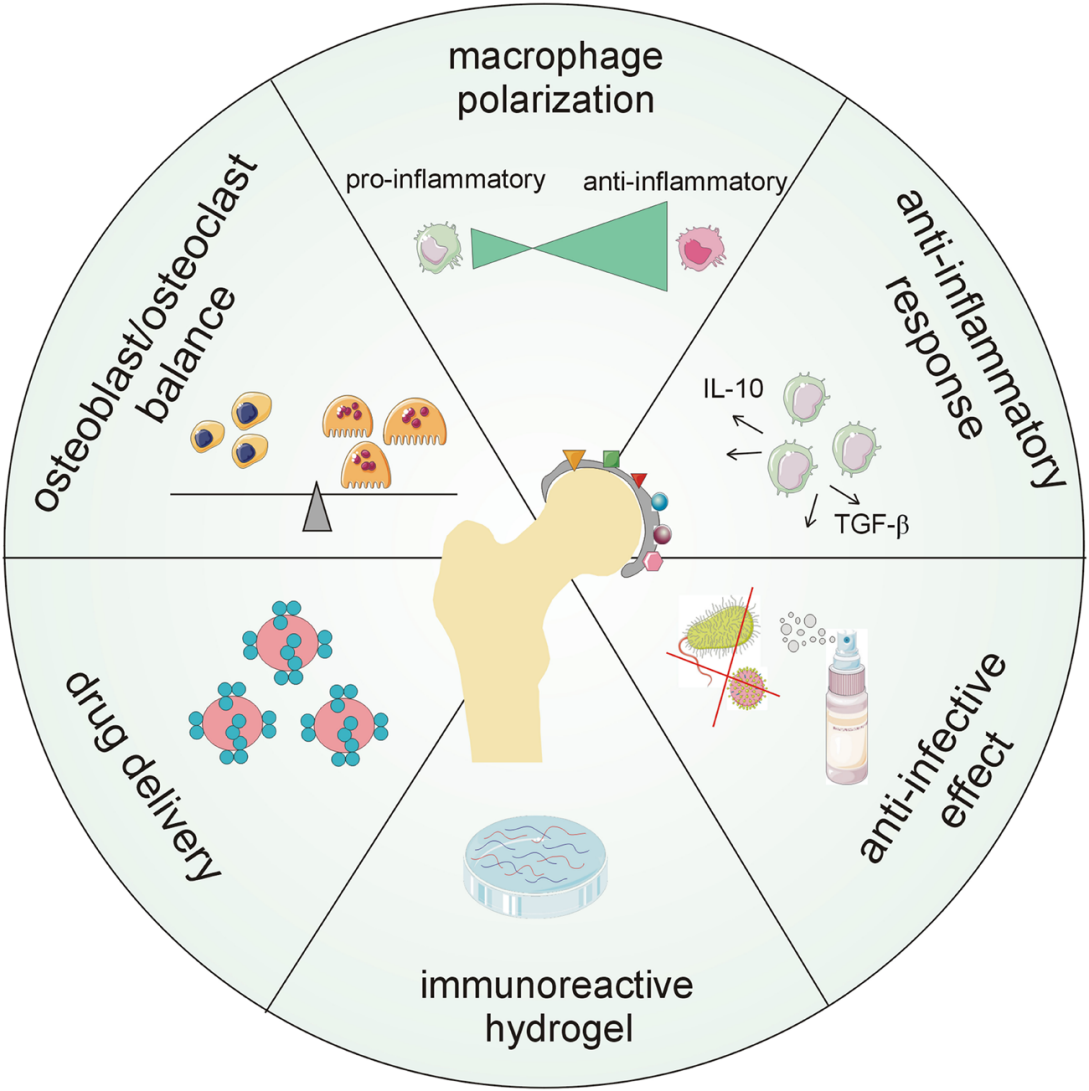
Major aspects demanded of new generations of biomaterial surfaces including chemokines. The future goal is to create multi-functional coatings that will provide long-term protection of orthopedic implants in terms of antimicrobial, anti-biofilm, and anti-inflammatory capability, modulation of host cellular response, tunable drug loading, and controlled and localized delivery of therapeutics.

An appropriate biomaterial should include “regenerative” surface modification ensuring macrophage polarization and long-term anti-inflammatory and anti-bacterial properties. Moreover, it may include nano- and micro-particles in biomaterial composites delivering active drugs or modifiers and/or coatings with immunoreactive components (Figure 4).

Homeostatic surface treatment for biomaterials should be designed to interfere with pro-inflammatory and pro-osteolytic mechanisms running around a TJR. Implant fixation surfaces could be treated with a combination of structural modifications and a mix of bioactive substances (including cytokines/chemokines). The latter should affect the number/survival of osteoblasts, as well as inflammation and osteoclast maturation. In addition, these substances should be released in a controlled manner and specifically with regard to individual tissue requirements (on demand) to eliminate detrimental side effects. Since there is a growing body of evidence on the crucial role of macrophages, especially M2 “tissue healing” macrophages, in the integration of implanted biomaterials (103–106), drug-eluting implants, designed to control macrophage behavior and attraction, may be used to control and tune the endogenous repair processes (95–97). Additionally, chemokines, inhibitory mutant chemokines, antagonists of chemokine receptors, or antibodies to the chemokine system may be incorporated into multi-functional surface layers and may contribute to the control of cell recruitment and polarization of macrophages, as well as to the stimulation of microvascular network remodeling shortly after implantation. Several studies on animal models also showed the potential of using cytokines/chemokines to prevent infection associated with the implant or fracture healing (107–109). Moreover, there is a growing body of evidence about the direct and rapid influence of implant surface treatments on the modulation of the expression of chemokine receptors that are important for cell recruitment and adhesion, both processes being crucial for the inflammatory and regenerative processes *in vivo* (110, 111). An appropriate implant surface may attenuate the inflammatory response while enhancing mineralization during osseointegration, as shown for implants with nano-surfaces (112) or the synthetic lipid polymer 2-methacryloyloxyethyl phosphorylcholine (113).

Currently, there is still limited information on the best combination of agents (cytokines/chemokines being promising candidates) for short- and long-term protective and supportive campaigns working at the implant–bone interface and its surroundings. In addition, the implant should exhibit anti-bacterial behavior, at least early post-operatively. Thus, further research is required to develop multi-functional implant surfaces, including chemokines, possessing antimicrobial, anti-biofilm, and anti-inflammatory capability as well as controlled and localized delivery of therapeutics (Figure 4).

## Conclusion and Prospects

The chemokine system relevant to the context of orthopedic implant debris is mainly involved in the migration of macrophages and osteoclasts to the site around implants, apoptosis, angiogenesis, collagen production, and tissue remodeling, which act together to elicit PPOL/AL. Generally, the role of the chemokine system in human PPOL/AL is underestimated and not well understood. Most of our current understanding of this system in PPOL/AL comes from *in vitro* models or animal studies that may be overly simplistic compared to the human situation. There is evidence of the need to introduce a different comprehensive manner of investigation enhancing the knowledge of the tangled chemokine network contributing to the osteolytic process.

Recent studies on murine models of PPOL showed the potential offered by targeting the CCR2–CCL2, CXCR2–CXCL2, and CXCR4–CXCL12 interactions in mitigating osteolytic processes, suggesting chemokine receptor–chemokine interaction as a potential therapeutic target in preventing implant failure. However, the complexity and redundancy of the chemokine system indicate that the interruption of a single, albeit potent, chemokine receptor–chemokine interaction is unlikely to succeed clinically without a more sophisticated understanding of this interplay. In contrast, auspicious results arising from the latest studies showed a possible use of implant surface coatings incorporating chemokines, inhibitory mutant chemokines, antagonists of chemokine receptors, or neutralizing antibodies to the chemokine system, thus promoting tissue regeneration *via* macrophage polarization and the regulation of adhesion, as well as controlling inflammation and preventing infection. All these processes might lead to lower rates of complications accompanying TJRs. Nevertheless, further studies on the appropriate modification of implant surfaces are highly desirable; the quest for a surface biomaterial with anti-inflammatory and anti-bacterial properties, minimizing the continual attack of pro-osteolytic agents and delivering active drugs according to specific tissue requirements, remains a challenge for future exploration.

Taken together, these findings suggest that strategies that interfere with cell recruitment and tissue response through chemokine signaling may modulate the adverse reaction to orthopedic implants and their by-products. Technologies, for example, bioactive orthopedic implant coatings, may play a role in improving the survival of TJRs by modulating cell trafficking to the bone–implant interface and changes in the tissue response.

## Author Contributions

TD drafted the manuscript and prepared the figures. EK conceived and designed the study and drafted the manuscript. JG edited and revised the manuscript. TD, EK, and JG approved the final version of the manuscript.

## Conflict of Interest Statement

The authors declare that the research was conducted in the absence of any commercial or financial relationships that could be construed as a potential conflict of interest.
